# Painful ulnar artery leiomyoma simulating an ulnar artery aneurysm

**DOI:** 10.1590/1677-5449.202500622

**Published:** 2025-11-03

**Authors:** Srijita Saha, Utkarsh Anand, Gokulakrishnan Hari, Varsha Khandelwal, Ujjwal Gorsi, Debajyoti Chattergi, Ajay Savlania

**Affiliations:** 1 Postgraduate Institute of Medical Education and Research – PGIMER, Chandigarh, India.

**Keywords:** ulnar artery, ulnar artery aneurysm, leiomyoma, artéria ulnar, aneurisma da artéria ulnar, leiomioma

## Abstract

Angioleiomyomas are rare, benign, smooth muscle tumors that occur predominantly in the lower limbs and infrequently in the upper limbs. We present a case of angioleiomyoma arising from the distal ulnar artery, presenting as a painful lesion with a 20-year history of diagnostic uncertainty. It was preoperatively misdiagnosed as an ulnar artery aneurysm. Surgical excision with primary end-to-end arterial repair was performed. Histopathological examination confirmed features consistent with a leiomyoma arising from the distal ulnar artery wall. The patient became asymptomatic following the surgery and remains pain-free at 6 months of follow-up.

## Resumo

Os angioleiomiomas são tumores benignos raros que se originam do músculo liso. Acometem predominantemente os membros inferiores e, mais raramente, os superiores. Relatamos o caso de um angioleiomioma originado na artéria ulnar distal, manifestando-se como uma lesão dolorosa com histórico de 20 anos de incerteza diagnóstica. Inicialmente, a lesão foi erroneamente diagnosticada como um aneurisma da artéria ulnar. O tratamento consistiu em excisão cirúrgica, seguida de reparo primário término-terminal da artéria. O exame histopatológico confirmou características compatíveis com leiomioma originado na parede da artéria ulnar distal. Após a cirurgia, o paciente evoluiu sem sintomas e permanece sem dor após 6 meses de acompanhamento.

## INTRODUCTION

First described in 1937, an angioleiomyoma, also known as angiomyoma, vascular leiomyoma, or dermal angioma, is a benign smooth muscle tumor that arises from the tunica media layer of subcutaneous vessel walls.^1^ It accounts for 4.4 percent of all soft tissue tumors.^2^ Although it can occur throughout the body, the most common site is the lower limb, with the rarest location being the hand.^3^ The majority arise from veins; only a few cases originate from arteries, and tumors causing nerve compression with associated neuropathic symptoms are even rarer.^4^ The rarity of this condition often leads to misdiagnosis. No diagnostic modality other than biopsy can confirm diagnosis, because it lacks distinctive clinical or radiological features.^3^ Local surgical excision is usually curative and associated with excellent prognosis.^5^ An extremely rare case of malignant transformation has also been reported.^6^

### Ethics statement/ethical approval

Ethical approval was not required for this case report, as per the policy of the Institutional Ethics Committee. The study complies with the ethical standards laid down in the 1964 Declaration of Helsinki and its subsequent amendments. Written informed consent was obtained from the patient for publication of the case details and accompanying images. A copy of the written consent is available from the corresponding author.

## CASE REPORT

A 63-year-old woman presented with pain over the ulnar aspect of the right wrist for 20 years. The pain had insidious onset and was gradually progressive. Initially occurring once every two months, frequency later increased to one to two episodes per day. The pain was intermittent, described as a current-like sensation, initially mild but later severe in intensity, lasting 20 to 30 minutes per episode. It was unresponsive to oral analgesics and disturbed her sleep at night.

On local examination, there was a 1 × 1 cm soft swelling on the volar aspect of the ulnar side of the wrist. It was non-tender, with no overlying skin changes or neurovascular deficits. Both radial and ulnar arteries were palpable on the right side. Basic blood investigations, including C-reactive protein, were within normal limits. Autoimmune markers, including cANCA and pANCA, were negative.

**Figure 1:**
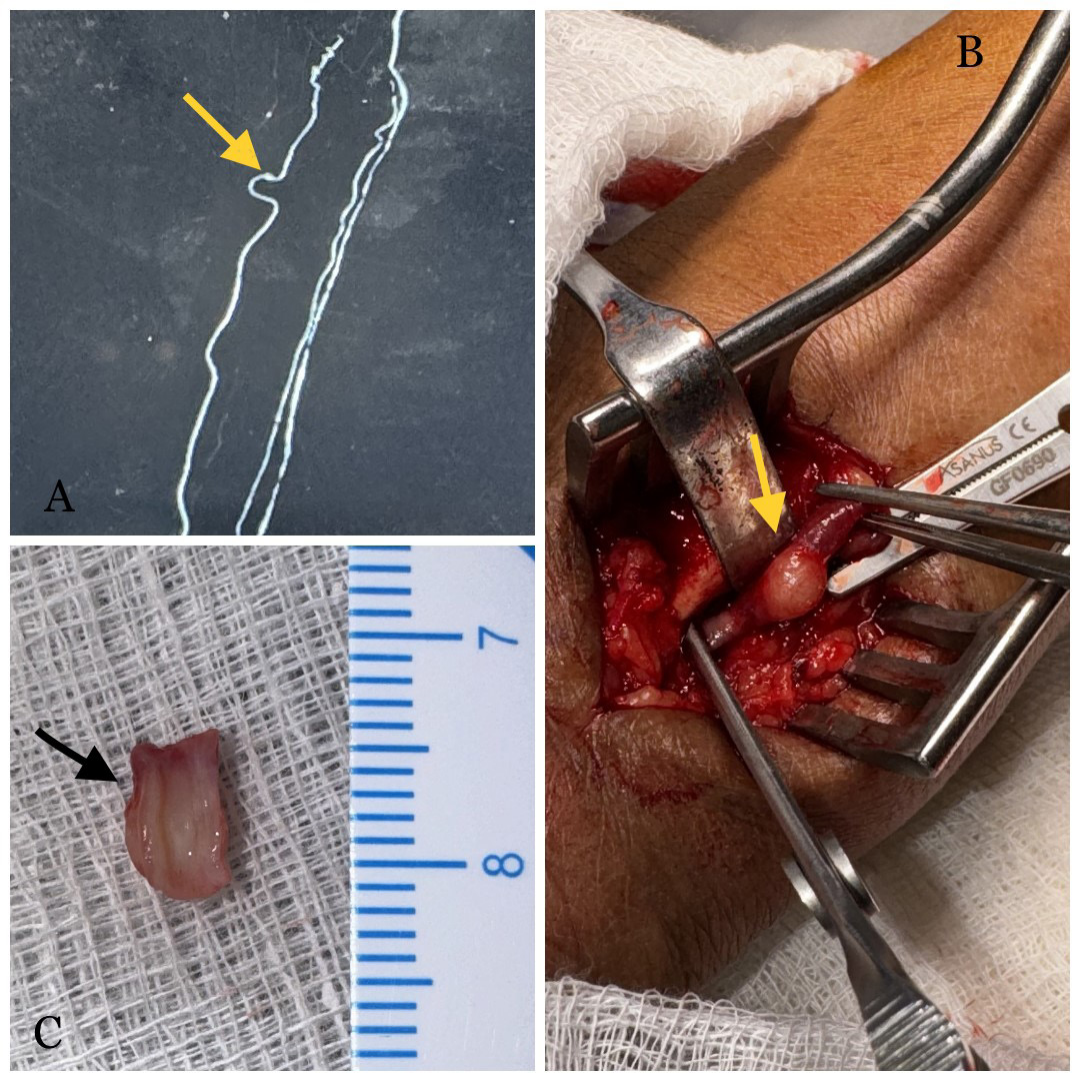
(A) CT angiogram with volume rendering of the right forearm region showing a kink in the ulnar artery with aneurysmal dilatation of the ulnar artery (arrow); (B) an intraoperative image showing aneurysmal dilatation of the ulnar artery with proximal and distal segments clamped, followed by excision and repair (arrow); (C) the excised aneurysmal segment of the ulnar artery, with the cut section revealing a healthy intima within the excised segment (arrow).

Ultrasound revealed normal thickness of both the median and ulnar nerves. Nerve conduction studies were also normal. MRI of the right wrist demonstrated a minimal STIR hyperintense soft tissue lesion suggestive of a ganglion cyst, measuring 4.7 × 2.8 cm, along the central aspect of the trapezium; no enhancement was seen in the arterial phase. CT angiography showed normal right subclavian, axillary, and brachial arteries, with a 5 × 3 mm dorsolaterally opacified fusiform dilatation of the distal right ulnar artery just proximal to the wrist joint, suggestive of a thrombosed pseudoaneurysm without calcification (Figure 1A).

The patient was planned for surgical excision and repair of the presumed pseudoaneurysm. Under local anesthesia, the ulnar artery was explored through a 4 cm longitudinal incision. A hairpin-shaped kinked segment of the ulnar artery was identified, with a 0.5 × 0.5 cm fusiform dilatation at the distal end of the kink (Figure 1B). After proximal and distal vascular control, a 1 cm segment including the kinked and aneurysmal portion was mobilized and resected. Primary end-to-end anastomosis was performed using 7-0 polypropylene. The cut section of the resected artery revealed a smooth and healthy intima, raising suspicion of a lesion involving the media layer (Figure 1C).

**Figure 2:**
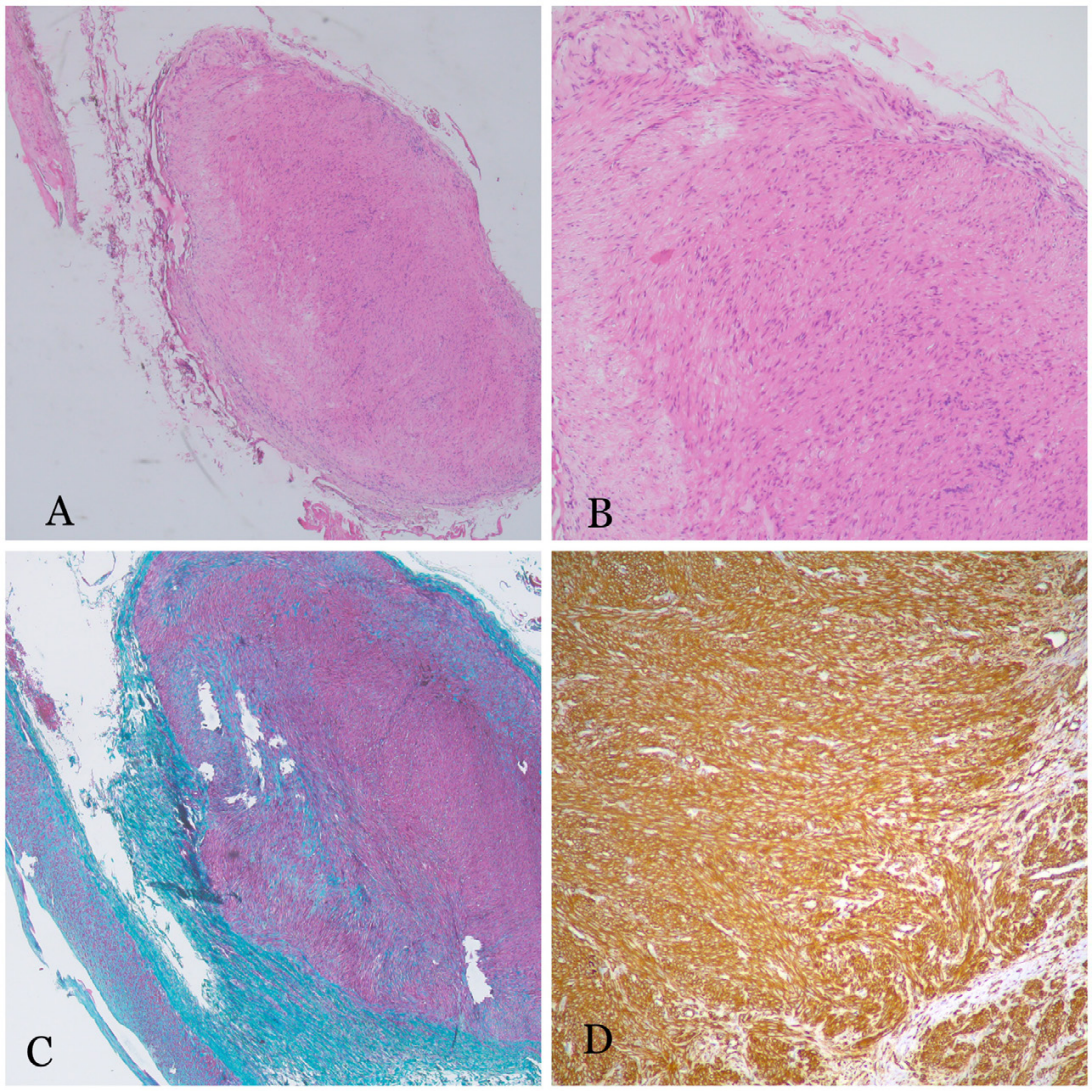
**(A)** histological examination shows part of a muscular artery with a nodule arising from its wall (Hematoxylin and Eosin, ×40); (B) the nodule is composed of bundles of smooth muscle cells with bland nuclei (Hematoxylin and Eosin, ×100); (C) Masson Trichrome stain shows that the nodule is composed of smooth muscle (×40); (D) the tumor cells show diffuse expression of smooth muscle actin (immunohistochemistry, ×200).

Histopathological examination confirmed a leiomyoma arising from the tunica media of the distal ulnar artery wall (Figure 2). The postoperative course was uneventful. At six months of follow-up, the patient remains pain-free and reports improved quality of life.

## DISCUSSION

Angioleiomyoma is a benign tumor arising from vascular smooth muscle, most commonly presenting between the third and fifth decades of life.^7^ The occurrence of vascular leiomyomas originating from arteries in the hand is rare, with only six cases reported in the English language literature.^3^ In the present case, the tumor was located in the palm and originated from the distal ulnar artery, approximately 3 cm proximal to the superficial palmar arch — an uncommon site for a vascular leiomyoma.

The exact etiology of angioleiomyomas remains unclear. Proposed contributing factors include minor trauma, hamartomatous changes, venous stasis, and hormonal imbalance.^5^ An arteriovenous malformation has also been reported in the literature and was noted in our case as well.^8^

Angioleiomyoma presents as a painful mass in approximately 60 percent of cases.^7^ One of the characteristic clinical features is enlargement of the swelling during physical activity of the affected part, particularly in the hand.^7^ In the present case, although pain was present, the swelling was small and did not demonstrate significant variation in size. Due to the associated current-like sensation suggestive of neuropathic pain, nerve conduction studies were performed. Ultrasound did not reveal any neural thickening. MRI raised a strong suspicion of a ganglion cyst. CT imaging showed saccular dilatation of the artery without contrast enhancement in the dilated segment, thus supporting the provisional diagnosis of a pseudoaneurysm. Blood tests were performed to rule out small vessel vasculitis.

The differential diagnosis includes schwannoma, neurofibroma, hemangioma, nodular fasciitis, pigmented villonodular synovitis, giant cell tumor of the tendon sheath, and true or false aneurysms.^9^ Definitive diagnosis requires histopathological examination.^5^ The standard treatment for symptomatic cases, such as ours, involves simple excision of the mass along with ligation of feeder vessels.^10^ It is essential to assess collateral circulation both preoperatively and intraoperatively. In cases where collateral flow is inadequate, vascular repair or grafting may be required. In our case, although collateral circulation was sufficient, we proceeded with primary end to end anastomosis of the ulnar artery.

Most angioleiomyomas of the hand are benign; however, malignant variants have been reported. If resection is complete, recurrence is rare, and prognosis is excellent.^5,6^

## CONCLUSION

For lesions arising from an artery, although a pseudoaneurysm is often the most common differential diagnosis, the possibility of angioleiomyoma should also be considered, as it is associated with excellent prognosis following resection and resolution of symptoms.

## Data Availability

Data not reported or used: “Data sharing does not apply to this article, as no data were generated or analyzed”.

## References

[1] Houdek MT, Rose PS, Shon W, Kakar S (2013). Angioleiomyoma of the upper extremity. J Hand Surg Am.

[2] Hachisuga T, Hashimoto H, Enjoji M (1984). Angioleiomyoma: a clinicopathologic reappraisal of 562 cases. Cancer.

[3] Miyamoto W, Yamamoto S, Kii R, Uchio Y (2008). Vascular leiomyoma resulting in ulnar neuropathy: case report. J Hand Surg Am.

[4] Enzinger FM, Enzinger FM, Weiss SW (1995). Soft tissue tumors..

[5] Shafi M, Hattori Y, Doi K (2010). Angioleiomyoma of distal ulnar artery of the hand. Hand.

[6] Hirata H, Kusuzaki K, Fukutome K, Maeda M, Uchida A (2005). A hand mass that became painful 13 years after onset. Clin Orthop Relat Res.

[7] Ramesh P, Annapureddy SR, Khan F, Sutaria PD (2004). Angioleiomyoma: a clinical, pathological and radiological review. Int J Clin Pract.

[8] Fox SB, Heryet A, Khong TY (1990). Angioleiomyomas: an immunohistological study. Histopathology.

[9] Callé SC, Eaton RG, Littler JW (1994). Vascular leiomyomas in the hand. J Hand Surg Am.

[10] Nagata S, Nishimura H, Uchida M, Hayabuchi N, Zenmyou M, Fukahori S (2006). Giant angioleiomyoma in extremity: report of two cases. Magn Reson Med Sci.

